# Cortical activity associated with focal muscle vibration applied directly to the affected forearm flexor muscle in post-stroke patients: an fNIRS study

**DOI:** 10.3389/fnins.2023.1281160

**Published:** 2023-12-13

**Authors:** Xianshan Shen, Yang Yu, Han Xiao, Leilei Ji, Jianxian Wu

**Affiliations:** ^1^Department of Rehabilitation Medicine, The Second Affiliated Hospital of Anhui Medical University, Hefei, China; ^2^Department of Rehabilitation and Sports Medicine, The Second Clinical College of Anhui Medical University, Hefei, China

**Keywords:** focal muscle vibration, stroke, cortical excitability, cortical activity, functional near-infrared spectroscopy

## Abstract

**Objective:**

The purpose of this study was to utilize functional near-infrared spectroscopy (fNIRS) to identify changes in cortical activity caused by focal muscle vibration (FMV), which was directly administered to the affected forearm flexor muscles of hemiplegic stroke patients. Additionally, the study aimed to investigate the correlation between these changes and the clinical characteristics of the patients, thereby expanding the understanding of potential neurophysiological mechanisms linked to these effects.

**Methods:**

Twenty-two stroke patients with right hemiplegia who were admitted to our ward for rehabilitation were selected for this study. The fNIRS data were collected from subjects using a block-design paradigm. Subsequently, the collected data were analyzed using the NirSpark software to determine the mean Oxyhemoglobin (Hbo) concentrations for each cortical region of interest (ROI) in the task and rest states for every subject. The stimulation task was FMV (frequency 60 Hz, amplitude 6 mm) directly applied to belly of the flexor carpi radialis muscle (FCR) on the affected side. Hbo was measured in six regions of interest (ROIs) in the cerebral cortex, which included the bilateral prefrontal cortex (PFC), sensorimotor cortex (SMC), and occipital cortex (OC). The clinical characteristics of the patients were assessed concurrently, including Lovett’s 6-level muscle strength assessment, clinical muscle tone assessment, the upper extremity function items of the Fugl-Meyer Assessment (FMA-UE), Bruunstrom staging scale (BRS), and Modified Barthel index (MBI). Statistical analyses were conducted to determine the activation in the ROIs and to comprehend its correlation with the clinical characteristics of the patients.

**Results:**

Statistical analysis revealed that, except for right OC, there were statistically significant differences between the mean Hbo in the task state and rest state for bilateral SMC, PFC, and left OC. A positive correlation was observed between the muscle strength of the affected wrist flexor group and the change values of Hbo (Hbo-CV), as well as the beta values in the left SMC, PFC, and OC. However, no statistical correlation was found between muscle strength and Hbo-CV or beta values in the right SMC, PFC, and OC. The BRS of the affected upper limb exhibited a positive correlation with the Hbo-CV or beta values in the left SMC and PFC. In contrast, no statistical correlation was observed in the right SMC, PFC, and bilateral OC. No significant correlation was found between the muscle tone of the affected wrist flexor group, FMA-UE, MBI, and Hbo-CV or beta values of cortical ROIs.

**Conclusion:**

FMV-evoked sensory stimulation applied directly to the FCR belly on the paralyzed side activated additional brain cortices, including bilateral PFC and ipsilesional OC, along with bilateral SMC in stroke patients. However, the clinical characteristics of the patients were only correlated with the intensity of ipsilesional SMC and PFC activation. The results of this study provide neurophysiological theoretical support for the expanded clinical application of FMV.

## 1 Introduction

Stroke is a widespread disease that endangers the lives and health of the middle-aged and elderly. According to the Global Burden of Disease (GBD) study, stroke continues to be the second main reason for mortality and the third cause of both mortality and disability (measured by disability-adjusted life-years lost-DALYs) in individuals (Feigin et al., 2022). After experiencing a stroke, roughly 26% of individuals continue to face disability in essential daily tasks, while half of them endure restricted mobility caused by hemiparesis (Kelly-Hayes et al., 2003). Stroke can induce altered muscle tone in the affected upper limbs and hands, decreased coordination, impaired sensation, muscle weakness, and impaired motor control, affecting activities of daily living and social participation. Scholars have commonly expressed their concern regarding how to enhance the effectiveness of limb function in patients with hemiplegic stroke. The main clinical treatments for post-stroke limb dysfunction include physical therapy (European and Rehabilitation Medicine Bodies, 2018), occupational therapy (Powell et al., 2016), transcutaneous electrical nerve stimulation (Mahmood et al., 2019), functional electrical stimulation (Eraifej et al., 2017), transcranial direct current stimulation (Van Hoornweder et al., 2021), repetitive transcranial magnetic stimulation (Starosta et al., 2022), motor imagery (Monteiro et al., 2021), virtual reality (Kim et al., 2020), constraint induced movement therapy (Rocha et al., 2021), brain-computer interface technology (Xue et al., 2021), and robotics (Bruni et al., 2018), etc. These intervention strategies have limitations due to the need for trained professionals, lack of precision, consensus evidence, and high implementation costs (Wang et al., 2020). Pursuing a safe and effective method for neurorehabilitation poses a significant and complex challenge.

Disruption of the sensory system after stroke plays an essential role in the motor dysfunction of hemiplegic limbs (Bolognini et al., 2016). The loss of proprioception can impact the correction of motor errors, and stroke often leads to the loss of tactile sensation, which can affect the control of limb movements (Hughes et al., 2015). The motor recovery process after stroke may be attributed to the ongoing reorganization of neural networks, and inducing plasticity in neural networks may be facilitated by activating cortical or thalamic circuits (Zhou et al., 2022). From this perspective, one of the most effective modulators of cortical motor and somatosensory structures is repetitive sensory input (Ward and Cohen, 2004). In recent years, many scholars have applied vibration therapy (VT) to stroke rehabilitation, and related studies have shown that vibration therapy is effective for post-stroke limb dysfunction and spasticity (Moggio et al., 2022). Two categories of VT are whole body vibration (WBV) and focal muscle vibration (FMV). FMV is a therapeutic method using a mechanical device to provide vibrational stimulation to the belly of a specific muscle or its tendons. Studies have concluded that it is advantageous in facilitating the restoration of limb motor function in stroke patients during both the acute (Toscano et al., 2019) and chronic phases of stroke (Marconi et al., 2011). However, there are currently no standardized protocols or regulations for using FMV. Additionally, there is still ongoing debate about its exact efficacy. Moreover, the specific mechanism of its application is yet to be fully understood, necessitating further clinical research and verification. Cortical activation and neural network remodeling are the internal recovery processes in most neurological diseases; the activation and reconstruction of neurocircuitry is the natural procedure to achieve functional recovery. Available evidence suggests that FMV acts as a powerful proprioceptive stimulus, which modulates brain and spinal cord plasticity for clinical therapeutic effects (Viganò et al., 2023). Several studies have explored the cortical activation changes in stroke patients in response to FMV. Positron emission tomography (PET), electroencephalography (EEG), and functional magnetic resonance imaging (fMRI) have been used to investigate the neural mechanisms of focal vibration in healthy individuals (Naito et al., 1999; Golaszewski et al., 2002; Naito, 2004; Casini et al., 2006; Lopez et al., 2017). There have been limited reports on cortical activation changes in stroke patients in response to FMV, particularly in real-time. The advancement of noninvasive functional brain imaging technology provides us with an essential tool for detecting various brain function dysfunctions. The detection of fMRI is limited because the focal vibration therapies used in the clinic are metal devices. Fortunately, functional near-infrared spectroscopy (fNIRS) is a cutting-edge method of imaging the brain’s activity, possessing various benefits such as being safe, noninvasive, portable, resistant to motion and electromagnetic interference, offering excellent spatial and temporal resolution, as well as enabling prolonged monitoring (Wang et al., 2023b). The main objective of this study was to use fNIRS to identify changes in cortical activity caused by FMV, which was directly administered to the affected belly of the flexor carpi radialis muscle (FCR) of hemiplegic stroke patients. Furthermore, the study sought to investigate how these changes relate to the clinical characteristics of the patients, ultimately improving our understanding of the possible neurophysiological mechanisms involved in these effects. Moreover, the efforts described above will help provide a dependable reference for developing optimal protocols for focal vibration therapy.

## 2 Materials and methods

### 2.1 Subjects

This study included 22 patients who had right hemiplegia after suffering a stroke and were admitted to the Second Affiliated Hospital of Anhui Medical University in 2022. The group consisted of 16 male and 6 female patients, with 18 of them having cerebral infarction and the remaining four having cerebral hemorrhage. Additionally, all patients were right-handed and had been suffering from the disease for a duration of 2 weeks to 1 year upon admission to the hospital (as shown in Table 1).

**Table 1:** Demographic and clinical characteristics of the patients.

Characteristics	Subjects (*N* = 22)
Age (years)^b^	63.05 ± 8.81
Gender, male/female^a^	16/6
Infarction/hemorrhage^a^	18/4
Dominant arm (right/left)^a^	22/0
Right hemiplegia/left hemiplegia^a^	22/0
Muscle strength (affected wrist flexor group)^b^	1.86 ± 1.32
Muscle tone (affected wrist flexor group)^b^	1.23 ± 0.92
BRS (affected upper limb)^b^	2.55 ± 1.22
FMA-UE (affected upper limb)^b^	16.59 ± 16.89
MBI^b^	55.45 ± 17.99

^a^The number of participants; ^b^Mean ± standard deviation.

### 2.2 Inclusion and exclusion criteria

The following criteria were used for inclusion: (1) Patients had to meet the stroke diagnostic criteria revised by the World Health Organization definition (Stroke, 1989) and undergo cranial CT and MRI to confirm the stroke; (2) Patients must be more than 2 weeks post-stroke onset; (3) Vital signs needed to be stable; (4) Patients must have the ability to sit for at least 30 min; (5) Patients and their family members had to agree to participate in this study. The following criteria were used for exclusion: (1) Severe cognitive impairment; (2) History of previous brain injury or complicating neurodegenerative diseases; (3) History of mental illness; (4) Presence of serious diseases affecting the liver, kidney, hematopoietic system, endocrine system, and osteoarthritis; (5) Peripheral vascular disease in the upper extremities such as deep vein thrombosis, vasculitis, Raynaud’s disease, etc.

### 2.3 Methods for assessing clinical characteristics

Professor Lovett’s 6-point scale (0–5), developed at Harvard University, United States, in 1916 assessed muscle strength. The subsequent information provides a brief overview of each level of muscle strength: Grade 0 indicates the absence of any muscle contraction. Grade 1 denotes the presence of slight muscle contraction without resulting in joint movement. Grade 2 suggests the ability to generate some muscle movement, albeit not against the force of gravity. Grade 3 signifies the capability to counter mild resistance, though incapable of overcoming significant resistance. Grade 4 denotes the capacity to counter moderate resistance, yet insufficient to completely overcome maximum resistance. Finally, Grade 5 signifies combatting maximum resistance and maintaining normal muscle strength (Hidayat et al., 2015).

Muscle tone was evaluated on a scale ranging from grade 0 (low) to grade 4 (severely increased), with grade 1 indicating normal muscle tone, grade 2 indicating slightly increased muscle tone, and grade 3 indicating greater muscle tone.

The upper extremity function items of the Fugl-Meyer Assessment (FMA-UE) and the Bruunstrom Recovery Stage (BRS) were employed to assess the severity of post-stroke paralysis. FMA-UE is composed of 9 main items and 33 subitems, with each subitem graded on a scale of 0, 1, or 2. This results in 66 points, wherein higher scores indicate superior upper extremity function (Fugl-Meyer et al., 1975). BRS is a model that linearly describes the process of motor recovery in six different stages, each representing a different motor pattern and muscle control, and the motor recovery of most stroke patients after the disease onset conforms to the BRS pattern (Huang et al., 2016).

The Modified Barthel Index (MBI) was used to measure activities of daily living, which included 10 activities: eating, bathing, grooming, dressing, bed and chair transfer, ambulation (walking, wheelchair use), stair walking, bowel control, and urinary control. Each activity was rated on a graded scale, ranging from fully independent to entirely dependent, and the total score was 100.

### 2.4 Measurement of fNIRS

#### 2.4.1 Probe positioning and measurement points

**Figure 1:**
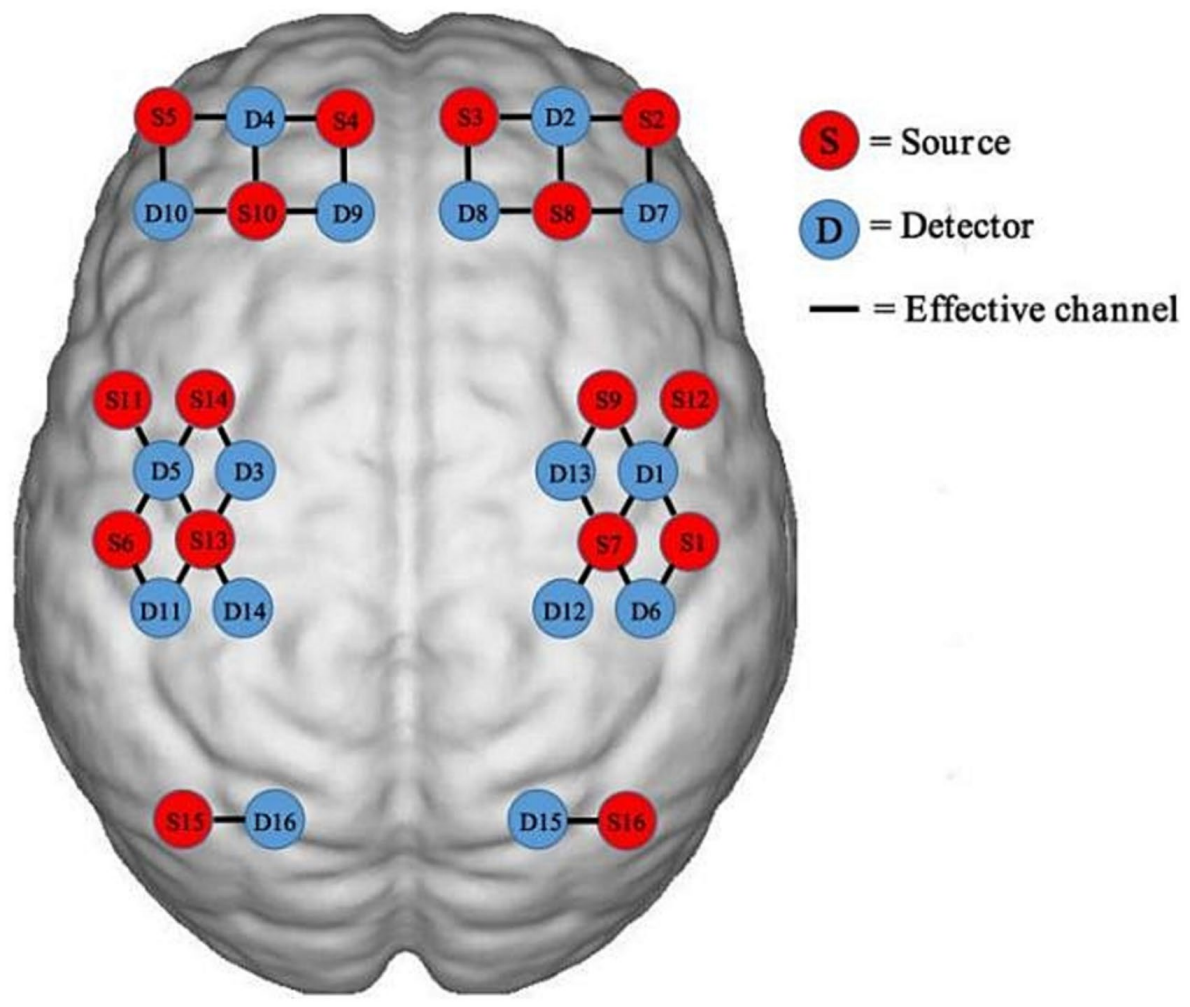
Functional near-infrared spectroscopy (fNIRS) probe positioning and measurement points.

For this study, a fNIRS system called Nirscan-6000A, manufactured by Danyang Huichuang Medical Equipment Co., Ltd. in China, was used to detect changes in Hbo signals. This device has been previously used in previous studies (Deng et al., 2022; Lin et al., 2022; Ma et al., 2023). The sampling rate for this study was set at 11 Hz, and the wavelengths used were 730 nm, 808 nm, and 850 nm. The system’s design included 32 probes, consisting of 16 sources and 16 detectors, following the 10/20 international standard line system. 34 channels were present in these probes, covering the cortical regions of interest (ROIs). The left and right prefrontal cortex (PFC) had seven channels each, while the left and right sensorimotor cortex (SMC) had nine channels each. Each left and right occipital cortex (OC) had one channel, as shown in Figure 1.

#### 2.4.2 Activation task

The fNIRS data was collected using a block-design paradigm. The stimulation task involved FMV of the flexor carpi radialis in the upper limb of the affected side (Celletti et al., 2020), which was administered using a deep muscle stimulation therapy device (ZEPU-K5000A, ZEPU Medical Equipment Co. Ltd., China), with the stimulation position located at the muscle belly of the flexor carpi radialis on the affected side. The stimulation was carried out at 60 Hz and an amplitude of 6 mm.

The fNIRS test was conducted in a quiet room with only the experimenter and participant present. The participant was instructed to ensure they had enough sleep and were in a normal mental state before the test. They were also instructed to avoid consuming stimulating foods and drugs, such as tobacco, alcohol, tea, and coffee. The participant was seated in a bent-legged position, with their upper limbs naturally resting on soft pillows. Their elbows were bent, and their forearms were rotated outward. They were instructed to remain relaxed, not to move their head or speak, and to minimize distractions. The tester used simple and accurate verbal cues to initiate and conclude each task. The subjects received FMV on the affected FCR in the following order (block-design paradigm): 30 s of FMV followed by 30 s of rest. This pattern continued for 5 cycles. Throughout both the task and rest states, the fNIRS device was used to measure Oxyhemoglobin concentrations (hereinafter referred to as Hbo) in all channels.

#### 2.4.3 Data analysis of fNIRS

This research used the NirSpark software to analyze the fNIRS data. Data preprocessing involved six steps: eliminating experimentally irrelevant time intervals, removing artifacts unrelated to the experimental data, transforming light intensity to luminous density, choosing band-pass filters (0.01–0.2 Hz) to filter out the noise and interfering signals, changing luminous density to blood oxygen concentration, and configuring the initial time of the hemodynamic response function (HRF) to −2 s and the end time to 60 s. The retained baseline state was from −2 s to 0 s, while the time of the single block paradigm was from 0 s to 60 s. Under the task state time setting of 30 s, the blood oxygen concentrations of the five blocking paradigms were superimposed and averaged to produce a blocking average result. The time-series data of Hbo was analyzed using a generalized linear model (GLM). The data was preprocessed for each channel and each subject. Subsequently, a t-test was conducted to compare the baseline and task statuses, with a significance level set at *p* < 0.05. The GLM facilitated the generation of an ideal hemodynamic response function (HRF) for each task. Following this, a comparison was made between the experimental and ideal HRF values to determine the corresponding range. The beta value, which shows the extent of cortical activation, served as an indicator for estimating the HRF prediction of the Hbo signal (Kawabata Duncan et al., 2019).

All participants completed the clinical scale assessment and fNIRS testing within 48 h of admission. Before completing the assessment, subjects had not received any rehabilitation therapy to ensure that such treatment did not influence the results. A highly skilled professional physiotherapist conducted the clinical scale assessment, while the fNIRS test was administered by a systematically trained master’s student.

### 2.5 Statistical analysis

The statistical analysis of the data was conducted using IBM SPSS (v.26.0). In order to examine the effects of cortical activity induced by FMV, t-tests were used to analyze the difference between the mean Hbo values of the task state and rest state in each cortical region of interest (ROI) at the group level. To determine if the sample data adhered to a normal distribution, the Shapiro–Wilk test was used. If the data followed a normal distribution, the paired samples t-test was utilized for statistical analysis to derive the *T*-value. On the other hand, if the sample data did not conform to a normal distribution, the Wilcoxon signed-rank test was applied to derive the Z-value. To gain further insight into the relationship between cortical activity and clinical characteristics, the change values of Hbo (Hbo-CV) in the task and rest states for each cortical ROI, along with the beta values and the patient’s clinical assessment data, were analyzed using Pearson’s correlation analysis or Spearman’s correlation analysis. Pearson correlation analysis was employed for sample data conforming to a normal distribution, while for sample data not conforming to a normal distribution, Spearman correlation analysis was used. A significance level of *p* < 0.05 was regarded as statistically significant.

### 2.6 Protocol approvals

The study was conducted at the Department of Rehabilitation Medicine of the Second Affiliated Hospital of Anhui Medical University. The Ethics Committee of the Second Affiliated Hospital of Anhui Medical University approved the experimental methods (approval number YX2022-018F1) and ensured compliance with the ethical standards stated in the 1975 Declaration of Helsinki, with revisions in 2008.

## 3 Results

### 3.1 Effects of cortical activity induced by FMV

**Figure 2:**
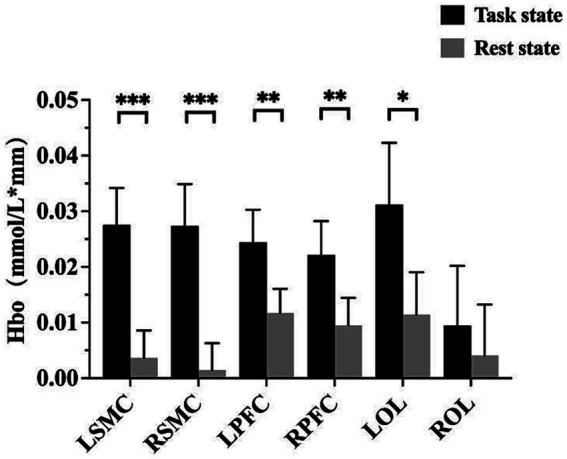
A bar chart with a summary date showing the activation of each cortical ROI. *p < 0.05; **p < 0.01; ***p < 0.001. LSMC, the left sensorimotor cortex; RSMC, the right sensorimotor cortex; LPFC, the left prefrontal cortex; RPFC, the right prefrontal cortex; LOC, the left occipital cortex; ROC, the right occipital cortex.

**Figure 3:**
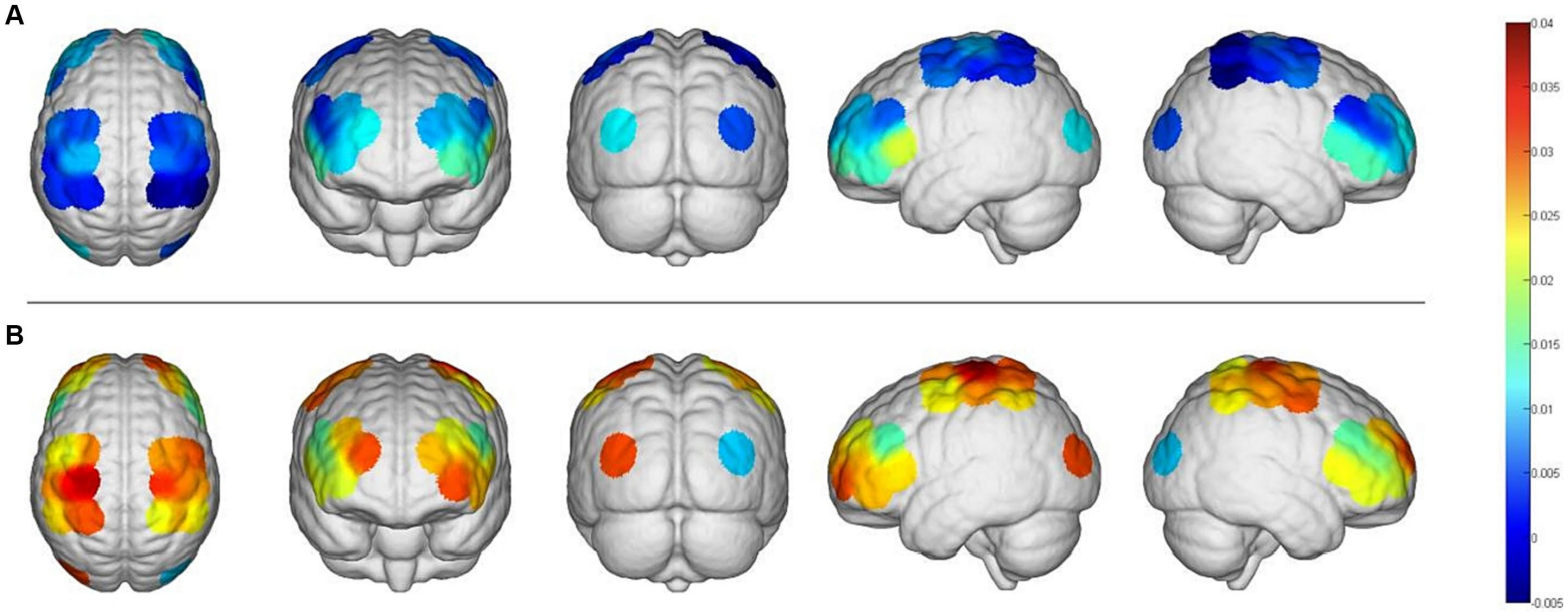
An anatomical view of cortical activation during two different phases, the task and the rest stages, is represented using different colors. **(A)**, Rest stage; **(B)**, task stage.

The fNIRS data were analyzed using the NirSpark software to capture the mean Hbo values for each cortical ROI in both the task and rest state for each subject. Statistical analysis indicated that, except for right OC, the differences between the mean Hbo in the task and rest states were statistically significant for bilateral SMC, bilateral PFC, and left OC. This suggests that the FMV treatment of the upper limb effectively activates the cortical regions of bilateral SMC, bilateral PFC, and left OC (Table 2; Figure 2). Figures 3A,B depict anatomical maps of cortical activation in two distinct phases, task state and rest state, at the group level. This visualization was plotted using the NirSpark software. Different colors on the images indicate these phases. The channel’s color corresponds to the mean Hbo level, with a redder tone indicating a higher mean Hbo and a bluer tone indicating a lower mean Hbo. Noteworthy color changes were observed in brain cortices such as bilateral SMC, bilateral PFC, and left OC, aligning with the findings of the aforementioned data analysis.

**Table 2:** The difference between the mean Hbo values of the task state and rest state in each ROI at the group level.

Cortical ROI	Hbo (task state)	Hbo (rest state)	*T*	*Z*	*P*
LSMC	0.0275 ± 0.0312	0.0037 ± 0.0231	5.59		0.000***
RSMC	0.0274 ± 0.0353	0.0015 ± 0.0226		−4.01	0.000***
LPFC	0.0244 ± 0.0274	0.0117 ± 0.0204	3.61		0.002**
RPFC	0.0222 ± 0.0284	0.0095 ± 0.0232	3.11		0.005**
LOC	0.0312 ± 0.0521	0.0114 ± 0.0356		−2.516	0.012*
ROC	0.0094 ± 0.0504	0.0041 ± 0.0431		−0.633	0.527

Bold indicates statistical significance (*P < 0.05; **P < 0.01; ***P < 0.001). Hbo (Task state), the average concentration of oxygenated hemoglobin in the task state; Hbo (Rest state), The average concentration of oxygenated hemoglobin in the rest state; LSMC, the left sensorimotor cortex; RSMC, the right sensorimotor cortex; LPFC, the left prefrontal cortex; RPFC, the right prefrontal cortex; LOC, the left occipital cortex; ROC, the right occipital cortex; *T*, *T*-value derived from paired *t*-test; *Z*, *Z*-value in nonparametric statistical tests.

### 3.2 Correlation between the cortical activity and clinical assessment data

**Figure 4:**
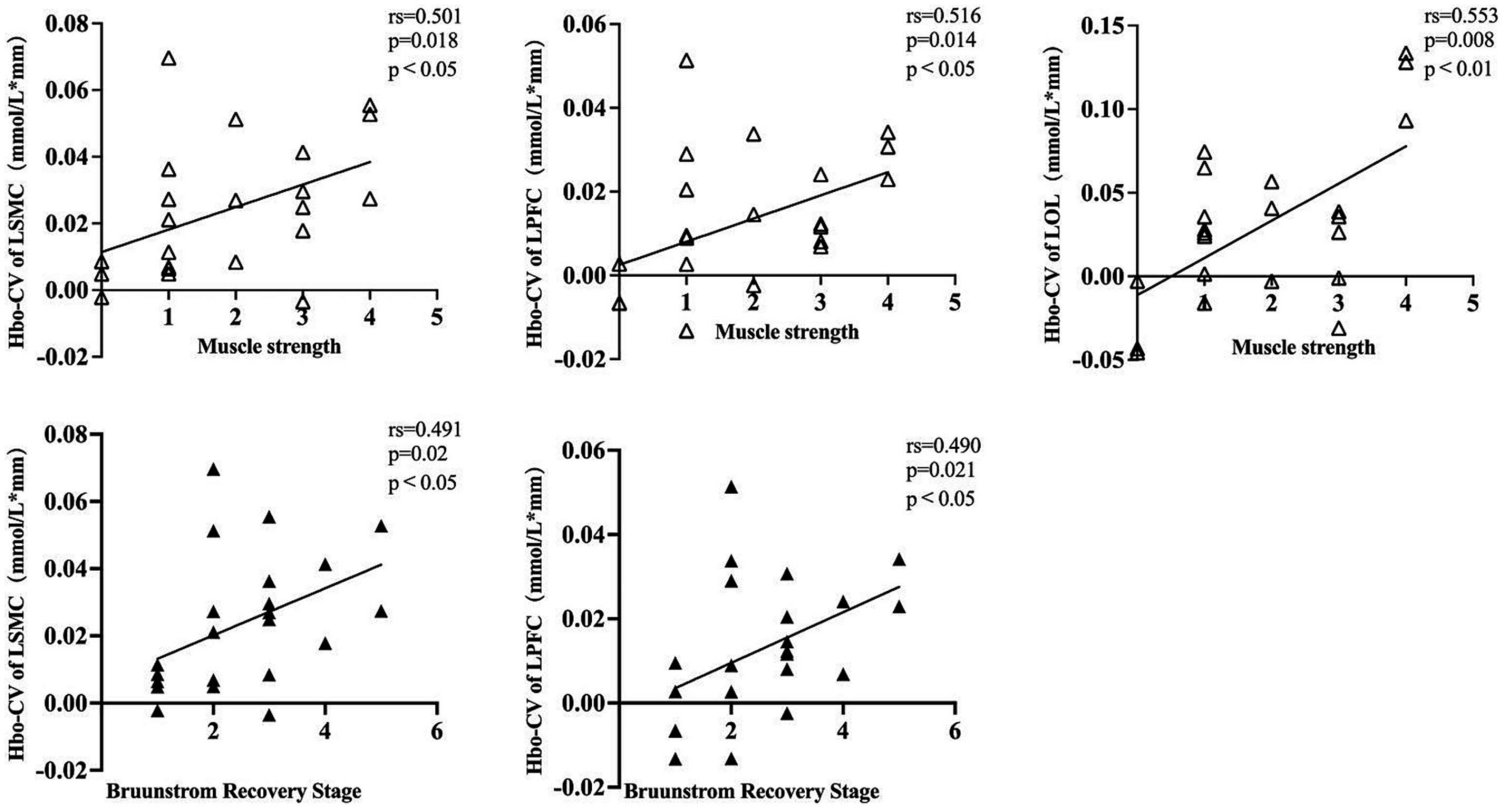
A dot plot showing the correlation between Hbo-CV and clinical assessment information for each cortical ROI. rs, Spearman correlation analysis correlation coefficient. LSMC, the left sensorimotor cortex; LPFC, the left prefrontal cortex; LOC, the left occipital cortex; muscle strength was assessed in the affected wrist flexor groups; Brunnstrom Recovery Stage was evaluated on the affected upper limb.

The Hbo-CV (change values of Hbo) in the task and rest states for each cortical ROI were calculated by subtracting the mean Hbo of the rest state from that of the task state. By analyzing the correlation between the Hbo-CV and the clinical assessment data, we can gain some insight into the association between the activation of different brain cortices and the patient’s clinical characteristics. Through Spearman correlation analysis, we found a positive correlation between the muscle strength of the affected wrist flexor group and the Hbo-CV in left SMC, PFC, and OC. However, no statistical correlation was found between muscle strength and Hbo-CV in the right SMC, PFC, and OC. The BRS (Bruunstrom Recovery Stage) of the affected upper limb showed a positive correlation with the Hbo-CV in left SMC and PFC, while no statistical correlation was observed in right SMC, PFC, and bilateral OC. No statistical correlation was found between the muscle tension of the affected wrist flexor group, FMA-UE, MBI, and the Hbo-CV of each cortical ROI (Table 3; Figure 4).

**Table 3:** The correlation coefficient between the Hbo-CV and clinical characteristics for each cortical ROI (*N* = 22).

	Hbo change value (Hbo-CV)					
	LSMC	RSMC	LPFC	RPFC	LOC	ROC
Muscle strength^a^	0.501*	0.392	0.516*	0.291	0.553**	0.249
Muscle tone^a^	0.298	0.247	0.24	−0.044	0.109	0.157
BRS^b^	0.491*	0.35	0.490*	0.335	0.397	0.081
FMA-UE^b^	0.256	0.139	0.334	0.143	0.238	−0.054
MBI	0.308	0.139	0.266	0.015	0.367	0.084

LSMC, the left sensorimotor cortex; RSMC, the right sensorimotor cortex; LPFC, the left prefrontal cortex; RPFC, the right prefrontal cortex; LOC, the left occipital cortex; ROC, the right occipital cortex; ^a^affected wrist flexor group; ^b^affected upper limb; Bold indicates statistical significance (*P < 0.05, **P < 0.01).

**Figure 5:**
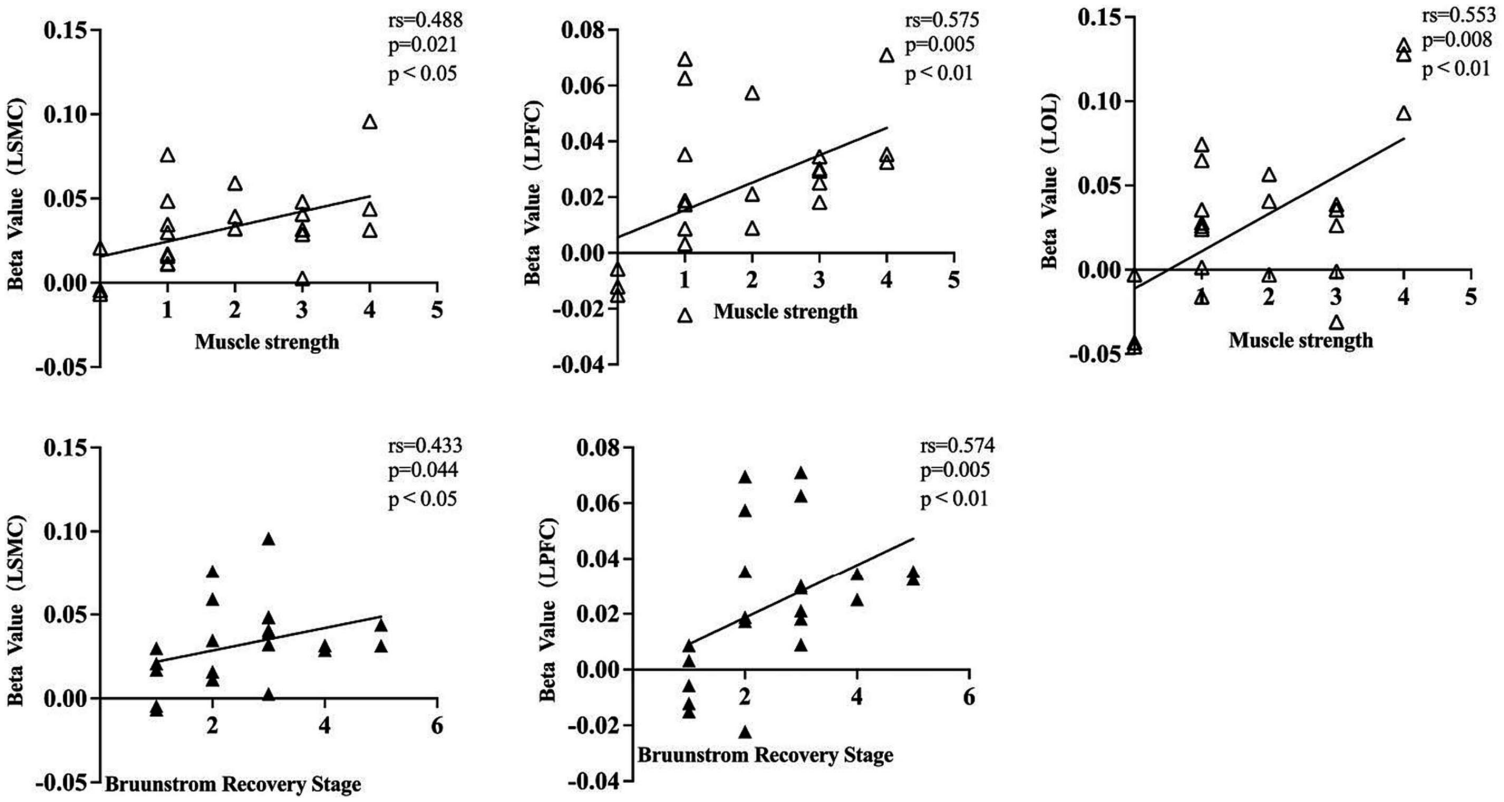
A dot plot showing the correlation between beta value and clinical assessment information for each cortical ROI. rs, Spearman correlation analysis correlation coefficient. LSMC, the left sensorimotor cortex; LPFC, the left prefrontal cortex; LOC, the left occipital cortex; muscle strength was assessed in the affected wrist flexor groups; Brunnstrom recovery stage was evaluated on the affected upper limb.

To gain a better understanding of the relationship between the patient’s clinical characteristics and the cortical activity produced by FMV, we conducted a Spearman correlation analysis using the beta values of each cortical ROI obtained from a GLM model and the clinic assessment. The results were consistent with the findings mentioned above. The muscle strength of the affected wrist flexor group showed a positive correlation with the beta values of left SMC, PFC, and OC. However, no statistical correlation was found in right SMC, PFC, and OC. The patient’s BRS on the affected upper limb exhibited a positive correlation with the beta values of left SMC and PFC. In contrast, no statistical correlation was found with the beta values of right SMC, PFC, and bilateral OC. Overall, no statistical correlation was observed between the patient’s affected wrist flexor group muscle tone, FMA-UE, MBI, and the beta values in each cortical ROI (Table 4; Figure 5).

**Table 4:** The correlation coefficient between the beta value and clinical characteristics for each cortical ROI (*N* = 22).

	Beta value					
	LSMC	RSMC	LPFC	RPFC	LOC	ROC
Muscle strength^a^	0.488*	0.286	0.575**	0.27	0.553**	0.249
Muscle tone^a^	0.326	0.15	0.332	0.112	0.109	0.157
BRS^b^	0.433*	0.224	0.574**	0.181	0.397	0.081
FMA-UE^b^	0.187	0.036	0.32	−0.03	0.238	−0.054
MBI	0.385	0.046	0.298	−0.114	0.367	0.084

LSMC, the left sensorimotor cortex; RSMC, the right sensorimotor cortex; LPFC, the left prefrontal cortex; RPFC, the right prefrontal cortex; LOC, the left occipital cortex; ROC, the right occipital cortex; ^a^affected wrist flexor group; ^b^affected upper limb; Bold indicates statistical significance (*P < 0.05; **P < 0.01).

## 4 Discussion

Several studies have used neurophysiological and neurofunctional imaging techniques, such as PET, fMRI, EEG, and TMS, to explore the neural mechanisms of focal vibration in healthy individuals. Naito et al. (1999) used PET scans to study the brain excitability of blindfolded subjects experiencing illusory arm movements through bicep tendon vibration. The study found significant excitability in contralateral motor areas and S1. (Golaszewski et al. (2002) used fMRI to study the cortical activity of healthy individuals during 50 Hz vibration of the right-hand palm. Results showed contralateral precentral and postcentral gyrus activation in all subjects, while some also exhibited ipsilateral activation. Naito (2004)) used fMRI to examine the cortical activity of focal vibration to produce a “motion illusion” in healthy subjects. The study found that the contralateral primary SMC, dorsal premotor cortex (PMd), supplementary motor area (SMA), cingulate motor cortex, and ipsilateral cerebellum were all activated. Casini et al. (2006) found that focal vibrating of the wrist tendons induced a “motor illusion” in healthy subjects, activating the contralateral primary SMC, SMA, and angular gyrus by using magnetoencephalography. Imai et al. (2014) used fNIRS to measure cortical activity during wrist tendon vibration at different frequencies in healthy subjects. They observed increased excitability in both cerebral hemispheres’ premotor cortex and parietal areas. An EEG-based study found that focal vibration applied to limb muscles modulated neurophysiological oscillations and increased contralateral S1-M1 excitability in healthy volunteers (Lopez et al., 2017). A randomized controlled study was conducted by Marconi et al. (2011) involving 30 hemiplegia patients who had suffered from a stroke. The experimental group, which received focal vibration stimulation in addition to physiotherapy, exhibited a lower resting motor threshold (RMT), an increased motor evoked potential (MEP) index, and an increased short-interval intracortical inhibition (SICI) compared to the control group, which only received physiotherapy. Another EEG study found that focal vibration (75 Hz) of the biceps brachii muscle on the affected side of subacute stroke can enhance contralateral S1-M1 excitability and alter the functional brain network (Li et al., 2019). An EEG study by Li et al. (2022) found that applying focal vibration (87 Hz, 0.28 mm) on the triceps of chronic stroke patients resulted in bilateral activation of the SMC. In summary, the majority of the studies mentioned above were conducted in healthy subjects due to the design of the study protocol and the experimental conditions, and the studies focused mainly on the SMC. Most of the studies found that focal vibration activated the contralateral sensorimotor cortex in healthy subjects, and there was only one EEG study (Li et al., 2022), which found that focal vibration activated the sensorimotor cortex bilaterally in stroke patients. The present study using fNIRS demonstrates that directly applying FMV on the affected FCR in stroke hemiplegic patients can immediately activate more brain cortex, such as the bilateral PFC and ipsilateral OC, in addition to the bilateral SMC. Based on the above findings, it is stated that hyperactivation of muscle proprioceptors induced by FMV can produce long-term potentiation (LTP) -like plastic changes that do not involve the sole ipsilesional sensorimotor cortex but probably entail a whole motor network relearning achieved through the plasticity-based modulation of the effective connectivity (Viganò et al., 2023).

Previous neuroimaging studies have reported contralateral brain activation in healthy subjects during active hand movements (Rao et al., 1993; Sabatini et al., 1993; Bonnal et al., 2023) In contrast, stroke patients more often experience increased excitability in both cerebral hemispheres during active movements of the affected hand (Marshall et al., 2000; Staudt et al., 2002; Rehme et al., 2011; Favre et al., 2014). The activation of bilateral sensorimotor areas induced by FMV was comparable to the results of some previous studies using fMRI or EEG which observed the activation of brain areas induced by motor imagery (Veverka et al., 2012), active movement (Veverka et al., 2014) and passive movement (Manganotti et al., 2010) as a task for the affected hand. Therefore, FMV, which acts as a potent proprioceptive stimulus, has some similar effectiveness to the activation of the brain produced by the movement-as-task paradigm. Previous studies on the activation of brain functions by FMV in stroke patients have rarely reported PFC and OC activation. This study showed that FMV applied to the flexor muscle of the forearm activated the bilateral PFC and the contralateral OC in addition to the bilateral SMC. Earlier studies have suggested that FMV applied to the limb can create “motor illusion” that can produce effective motor control and facilitate motor learning (Naito, 2004) (Imai et al., 2014). The PFC is an anterior region of the brain associated with cognitive control and executive functions (Friedman and Robbins, 2022). We hypothesize that the PFC may be involved in the perception, interpretation, and cognitive processes of the motion illusion produced by FMV to help understand how it is consistent with prior motion experience and intrinsic modeling. The OC is located in the posterior part of the brain and is primarily involved in the processing of visual information (Barton and Brewer, 2013). However, when FMV induces motion illusions, the OC may play a role in generating internal visual images, motion simulation, and pictorial motion illusions. Motion illusions induced by vibration can activate the processing of visual information in the occipital lobe, which in turn enhances and supports the perception of motion illusions.

Spasticity is one of the frequent complications after stroke, which affects the motor performance and rehabilitation process of stroke patients. The exact mechanism of post-stroke spasticity is not fully understood. Studies have shown that, among the downstream conduction tracts, the ventral medial reticulospinal tract from the pontine reticular formation and the vestibulospinal tract from the vestibular nucleus contribute to the increase in muscle tone; The dorsal reticulospinal tract from the bulbar reticular formation inhibited the excitability of the stretch reflex. The cerebral cortex modulates the stretch reflex by excitation of the bulbar reticular formation through the cortical reticular tracts (Trompetto et al., 2014; Naro et al., 2017; Bruni et al., 2018). The positive effect of FMV in reducing hemiplegic upper and lower limb spasticity in stroke patients has been confirmed by several reviews (Murillo et al., 2014; Alashram et al., 2019; Yang, 2020). A previous study applied FMV directly to the spastic muscles (Biceps brachii, Wrist flexor muscles, and Finger flexor muscles) of post-stroke hemiplegic patients and found that it was effective in decreasing spasticity of the target muscles as well as improving upper extremity function (Noma et al., 2009). The study discovered that vibratory stimulation resulted in a decrease in both the F-wave amplitude and the F/M ratio, which indicates a decline in motor neuron excitability. The current work discovered by fNIRS that FMV of the spastic muscle of the hemiplegic upper limb (wrist flexor muscle), leads to elevated cortical activity. The increase in sensorimotor cortical activation could indicate an improvement in inhibitory circuits, which helps to decrease spasticity (Vazquez et al., 2018). The possible mechanism is the aforementioned alteration of cortical activity, followed by excitation of the bulbar reticular formation through the corticoreticular bundle, as a result of a modulatory effect on the stretch reflex. The study found no correlation between the initial levels of muscle spasticity and the intensity of cortical activation induced by FMV. This suggests that there is no significant causal relationship between the level of muscle spasticity and the intensity of activation in the individual cerebral cortex following a stroke. Some articles suggest that FMV helps reduce spasticity by rebalancing interhemispheric interactions through the activation of bilateral S1-M1 (Li et al., 2022). Future longitudinal studies are necessary to better understand the relationship between FMV-induced spasticity relief and cortical activation when applied directly to the affected flexor muscles.

One previous meta-study found that the intensity of upper limb impairments in stroke patients was strongly linked to the level of activation in the ipsilesional M1 during movement of the paralyzed hand, as measured using fMRI (Rehme et al., 2012). The authors were inspired by the results of the above studies, and a search of the database did not reveal any relevant studies on the relationship between the activation of functional brain regions and movement performances in stroke patients as detected by fNIRS or fMRI studies using sensory stimulation as a task. In this study, we used fNIRS (a block-design paradigm) as a detection tool to perform a correlation analysis between the excitation intensity of cortical ROI and the clinical data of stroke patients using the FMV applied to the affected FCR (target muscle) as a task. The results revealed that the muscle strength grades of the target muscles were statistically positively correlated with the activation intensity in the ipsilesional ROIs, and not in the contralesional ROIs. The above results suggest that the muscle strength of the target muscles of the affected limb is closely related to the integrity of the sensory-motor nerve pathway and the functional status of the ipsilateral cortex of the lesion, which is also in accordance with the rules of traditional structural and functional anatomy. The outcomes of the ongoing study demonstrated that the BRS of the affected upper limb was positively correlated with the activation intensity of the ipsilesional SMC and LPFC, while it was not statistically correlated with the activation intensity of the contralesional SMC, PFC, and bilateral OC. The above results suggest that good activation of ipsilesional SMC and PFC induced by FMV-evoked sensory stimulation predicts a better motor recovery of the affected limb in stroke patients, which in part reflects the patient’s motor performance ability. This is somehow in line with the results of a previous meta-analysis that used fMRI and PET scans to study active or passive sensorimotor tasks involving the upper limb (Favre et al., 2014). A recent fNIRS study found that touching the affected shoulder in stroke patients can activate both sides of the motor cortex, the intensity of this activation was correlated with the baseline clinical characteristics of the patients. The authors attributed this result to functional compensation in uninjured brain regions (Zhang et al., 2023). This functional compensation is seen both within the affected hemisphere and between the unaffected hemispheres (Viganò et al., 2023; Zhang et al., 2023).

The design of an intervention program for focal vibration therapy mainly includes the settings of device parameters (such as frequency, amplitude, intensity, etc.), vibration duration, the targeted site for vibration action (such as flexor, extensor, muscle belly, tendon, etc.), and the varied timing in relation to stroke onset for recruited patients. it is widely reported that FMV’s capability of inducing synaptic plasticity using the proprioceptive pathway depends on vibration parameters that can deeply affect clinical results in RCT (Wang et al., 2020; Avvantaggiato et al., 2021; Viganò et al., 2023). Those aspects probably explain the absence of statistical correlations found in the present study between FMA-UE and MBI and the activation intensity of all cortical ROIs. Focal vibration stimulates the muscle spindle, and impulses are transmitted via Ia afferent fibers to alpha motor neurons and Ia inhibitory interneurons in the spinal cord. This afferent pathway produces an involuntary contractile response in the vibrated muscle, also known as a tonic vibration reflex (TVR), and a so-called “reciprocal inhibition” in the antagonist muscle, which inhibits spasticity in the antagonist muscle (De Gail et al., 1966; Hagbarth and Eklund, 1966). Therefore, there have been many previous studies of vibratory stimulation of antagonist muscles of spastic muscles to reduce spasticity in hemiplegic limbs (Bishop, 1975; Desmedt, 1983; Ageranioti, 1990; Casale et al., 2014; Constantino et al., 2014; Annino et al., 2019). Clinical observations have shown that vibration stimulation applied directly to spastic muscles produces an initial TVR in stroke patients, with significant relief of spasticity after several minutes of continuous stimulation (Noma et al., 2009). Hence, in recent years, many studies have used focal vibration to directly stimulate the spastic muscles (flexor muscles) in stroke patients, and have also achieved good efficacy (Noma et al., 2009; Marconi et al., 2011; Aprile et al., 2020). In recent years, the vibration frequency of local vibration applied to stroke rehabilitation ranges from 30 to 300 Hz, and the amplitude ranges from 0.01-10 mm (Wang et al., 2020, 2022; Viganò et al., 2023). It has been shown that vibrations of approximately 10 Hz or higher than 220 Hz do not produce a reliable and vivid “motion illusion.” Vibrations without the illusion of motion tend to activate the sensory areas of the brain, whereas vibrations with the illusion of motion activate the motor areas in addition to the sensory areas of the brain, and illusory limb movements activate the motor areas normally involved in executing and controlling limb movements (Naito, 2004). Previous studies have reported that in studies of vibration interventions for patients with spinal cord injury, multiple sclerosis, and other movement disorders, vibration amplitudes of 0.01 mm–2 mm, which were used for stroke rehabilitation, were comparable to vibration amplitudes of 0.005 mm to 10 mm (Murillo et al., 2014; Souron et al., 2017; Yang, 2020). Based on the above reasons, the present study adopted a treatment protocol of direct focal vibration (frequency: 60 Hz; amplitude: 6 mm) to the spastic muscles of the upper limb in stroke, using a therapeutic device (ZEPU-K5000A, ZEPU Medical Equipment Co., Ltd., China) that is widely used in neurological rehabilitation institutions in China, and which can induce “motion illusions” and TVR. In my clinical work, I found that applying this device to the flexor muscles of the hemiplegic upper limbs of stroke patients can reduce muscle spasticity and improve motor function of the upper limbs (ready for submission), without any side effects. A recent study using this same device in the treatment of hemiplegic stroke patients also showed that it was effective in improving upper limb motor function (Wang et al., 2023a). However, the decision on the selection of the above vibration parameters is still debated. It has been suggested that different vibration frequencies may preferentially activate different cortical areas (Chung et al., 2013; Avvantaggiato et al., 2021). Most literature currently uses vibration frequencies between 80 and 120 Hz, with 100 Hz being the most common. This frequency is believed to more easily induce plastic changes in the brain and spinal cord through cortical hyperactivity and motoneuron excitability, respectively; The optimal vibratory stimulus amplitude is <0.5 mm, as it activates Ia afferents via primary muscle spindle discharge. Higher amplitudes cause overflow, leading to TVR via amplified Ia inputs from persistent stimulation, which is still unclear if it is necessary for clinical effects (Viganò et al., 2023). Randomized controlled studies with different vibration frequencies and amplitudes are necessary in the future to further understand their effects on cortical activity and clinical function in stroke patients.

In addition, there are some other limitations of this study that can lead to bias in the results. First, the number of subjects was relatively small, the type of stroke (cerebral hemorrhage or cerebral infarction) was not restricted, and the exact location and size of the lesions were inconsistent, although the lesions were in the left cerebral hemisphere in all subjects. Therefore, more stroke patients must be recruited, the type of stroke needs to be limited, and the results can be better validated by controlling for the site and size of the injury in the patients. Second, adaptive neuroplasticity declines with time since acute stroke and improvement in clinical function peaks 3–6 months after stroke (Kwakkel and Kollen, 2013; Viganò et al., 2023). However, the duration of illness of the subjects recruited for this study ranged from 2 weeks to 1 year, which can also lead to bias in the results, so it would be preferable to recruit patients with acute or subacute stages of stroke as study subjects in future studies. Third, the current study used a block-design paradigm to detect changes in cortical activity, with the task being 30 s of FMV, which actually observed cortical activity during the FMV-induced TVR, not involving the cortical activity or brain networks after a TVR lasting several minutes, which needs to be added to future studies as well. Finally, assessment of functional activation of motor-related systems as an imaging biomarker has been suggested as a predictive method for prognosis after stroke (Zhang et al., 2022). The present study analyzed the correlation between FMV-induced changes in cortical activity and baseline clinical characteristics and did not perform a correlation analysis of changes in distant clinical characteristics. Therefore, further studies are necessary to confirm whether the prognosis of stroke patients can be predicted by analyzing FMV-induced changes in cortical activity.

## 5 Conclusion

The application of FMV-evoked sensory stimulation directly to the muscle belly of the FCR on the paralyzed side of the upper limb in stroke patients activated additional brain cortices, such as bilateral PFC and ipsilesional OC, in addition to bilateral SMC. This suggests that FMV induces neural plasticity across multiple brain regions. However, only the intensity of ipsilesional SMC and PFC activation showed a correlation with the clinical characteristics of the patients. The results of this study provide neurophysiological theoretical support for the expanded clinical application of FMV. Furthermore, the fNIRS test, which utilized a block-design paradigm of FMV, has the potential to be used as an objective assessment tool for hemiplegic stroke patients. Further studies are necessary to confirm whether the prognosis of stroke patients can be predicted by fNIRS detection of altered cortical activity induced by sensory stimuli evoked by FMV.

## Data availability statement

The original contributions presented in the study are included in the article/supplementary material, further inquiries can be directed to the corresponding author.

## Ethics statement

The studies involving humans were approved by the Ethics Committee of the Second Affiliated Hospital of Anhui Medical University. The studies were conducted in accordance with the local legislation and institutional requirements. The participants provided their written informed consent to participate in this study.

## Author contributions

XS: Conceptualization, Funding acquisition, Methodology, Writing – original draft, Writing – review & editing, Data curation, Formal analysis, Investigation, Project administration, Resources, Software, Supervision, Validation, Visualization. HX: Investigation, Writing – review & editing, Validation. LJ: Investigation, Project administration, Resources, Writing – review & editing. JW: Conceptualization, Methodology, Resources, Supervision, Writing – review & editing. YY: Data curation, Investigation, Software, Validation, Visualization.
